# Humanin skeletal muscle protein levels increase after resistance training in men with impaired glucose metabolism

**DOI:** 10.14814/phy2.13063

**Published:** 2016-12-06

**Authors:** Eva‐Karin Gidlund, Ferdinand von Walden, Mika Venojärvi, Ulf Risérus, Olli J. Heinonen, Jessica Norrbom, Carl Johan Sundberg

**Affiliations:** ^1^ Department of Physiology and PharmacologyKarolinska InstitutetStockholmSweden; ^2^ Neuropediatrics UnitDepartment of Women's and Children's HealthKarolinska Institutet and Astrid Lindgren's Pediatric HospitalStockholmSweden; ^3^ Institute of Biomedicine, Sports and exercise medicineUniversity of Eastern FinlandKuopioFinland; ^4^ Clinical Nutrition and MetabolismDepartment of Public Health and Caring SciencesUppsala UniversityUppsalaSweden; ^5^ Paavo Nurmi Centre and Departmen of Health & Physical ActivityUniversity of TurkuTurkuFinland

**Keywords:** Exercise, human, humanin, impaired glucose regulation, skeletal muscle

## Abstract

Humanin (HN) is a mitochondrially encoded and secreted peptide linked to glucose metabolism and tissue protecting mechanisms. Whether skeletal muscle HN gene or protein expression is influenced by exercise remains unknown. In this intervention study we show, for the first time, that HN protein levels increase in human skeletal muscle following 12 weeks of resistance training in persons with prediabetes. Male subjects (*n* = 55) with impaired glucose regulation (IGR) were recruited and randomly assigned to resistance training, Nordic walking or a control group. The exercise interventions were performed three times per week for 12 weeks with progressively increased intensity during the intervention period. Biopsies from the vastus lateralis muscle and venous blood samples were taken before and after the intervention. Skeletal muscle and serum protein levels of HN were analyzed as well as skeletal muscle gene expression of the mitochondrially encoded gene MT‐RNR2, containing the open reading frame for HN. To elucidate mitochondrial training adaptation, mtDNA, and nuclear DNA as well as Citrate synthase were measured. Skeletal muscle HN protein levels increased by 35% after 12 weeks of resistance training. No change in humanin protein levels was seen in serum in any of the intervention groups. There was a significant correlation between humanin levels in serum and the improvements in the 2 h glucose loading test in the resistance training group. The increase in HN protein levels in skeletal muscle after regular resistance training in prediabetic males may suggest a role for HN in the regulation of glucose metabolism. Given the preventative effect of exercise on diabetes type 2, the role of HN as a mitochondrially derived peptide and an exercise‐responsive mitokine warrants further investigation.

## Introduction

Regular exercise leads to adaptations such as mitochondrial biogenesis, improved oxidative capacity, and hypertrophy of skeletal muscle (Holloszy and Booth 1976). Aerobic and anaerobic training have also been recognized to improve glucose control in conditions such as impaired glucose tolerance and type 2 diabetes (Kelley et al. 2002; Earnest 2008). During the last decade, factors and molecules produced in skeletal muscle and secreted in response to exercise have attracted increased interest. The idea of exercise‐responsive factors, biologically active outside of the production site, has resulted in concepts such as myokines, exerkines, and exosomes being introduced (Pedersen et al. 2003; Safdar et al. 2016). These factors are diverse with respect to autocrine, paracrine, and/or endocrine function and the characterization of the human skeletal muscle secretome is still incomplete.

Improved metabolic control in persons with impaired glucose regulation (IGR) and in patients with type 2 diabetes (T2D) is plausibly mediated in part by restoration of impaired mitochondrial function and bioenergetics (Kelley et al. 2002; Meex et al. 2010). The complexity and function of the mitochondria are not yet fully understood, however, their role in organismal homeostasis and aging is undisputed (Bratic and Larsson 2013; Shadel and Horvath 2015). Human mitochondria contain a compact circular 16.5 kbp genome, with no known introns and very few noncoding nucleotides. Traditionally, the mitochondrial DNA (mtDNA) is considered to encode for only two rRNAs, 13 mRNAs, and 22 tRNAs. However, this view has recently been challenged. Mercer et al. reported unknown features of mitochondrial gene expression, function, and regulation which indicate that the mitochondrial transcriptome is far more complex than previously thought (Mercer et al. 2011). A specific example is the small mitochondrially encoded and secreted peptide humanin (HN), which is a 24‐amino acid polypeptide first identified and described in a cDNA library generated from a human Alzheimer's disease brain (Hashimoto et al. 2001a). HN is transcribed from an open reading frame (ORF) within the mitochondrially encoded 16S rRNA gene, that is, a gene within a gene. Since its initial discovery in humans, several cDNAs sharing sequence homology to HN have been identified in, for example, plants and rodents demonstrating that HN is evolutionarily conserved (Maximov et al. 2002; Bin Guo et al. 2003).

HN has been described as a neuroprotective and antiapoptotic factor (Hashimoto et al. 2001b; Bodzioch et al. 2009). However, the role and function of HN is complex and several other features have been coupled to HN activation, such as improved beta cell function and peripheral insulin signaling (Muzumdar et al. 2009). Furthermore, the HN analog HNGF6A has been shown to increase glucose‐stimulated insulin secretion in both normal and diabetic mice (Kuliawat et al. 2013). Interestingly, Voigt and Jelinek recently showed that HN levels in plasma are lower in patients with elevated fasting glucose compared to a control group (Voigt and Jelinek 2016).

Since HN has been suggested to play an important role in cellular homeostasis, and because plasma levels of HN are lower in individuals with impaired glucose control, it is relevant and of interest to investigate whether regular exercise affects skeletal muscle and serum expression of HN in this population. Accordingly, we investigated whether 12 weeks of training in men with impaired glucose regulation (IGR) could be enough to elicit a change in HN levels and if the response differed between resistance and endurance‐type training. We hypothesized that skeletal muscle and serum HN protein levels would be exercise‐responsive and increases with training in men with IGR.

## Materials and Methods

### Ethical approval

The study was approved by the Coordinating Ethical Committee of the Hospital District of Helsinki and Uusimaa. All subjects gave their written informed consent before participating. The study conformed to the standards set by the Declaration of Helsinki.

### Subjects and training protocol

The included study participants (*n *=* *55) constitute a subgroup from a larger randomized intervention study (Venojärvi et al. 2013a,b; Wasenius et al. 2014). The participants were recruited through occupational healthcare services located in Southern Finland and through newspaper advertisements. A total of 267 subjects were screened for eligibility. Suitable subjects (*n *=* *144) were equally randomized (1:1:1) into the intervention groups, resistance training (RT) or Nordic walking (NW) and a control group (C). A total of 115 subjects completed the study and from them a subgroup of 55 subjects (RT *n *=* *20, NW *n *=* *18, C *n *=* *17) donated muscle samples. The criteria for inclusion were as follows: male, aged 40–65 years, passed medical examination, elevated risk of type 2 diabetes (>12 points on the Finnish diabetes risk score (Lindström and Tuomilehto 2003)), newly assessed IGR (defined as fasting plasma glucose 5.6–6.9 mmol/L and/or 2 h plasma glucose 7.8–11 mmol/L after an oral glucose tolerance test), body mass index (BMI) 25.1–34.9 kg/m^2^ and no other metabolic diseases affecting glucose balance. Exclusion criteria were the following: previous detection of impaired glucose tolerance (IGT) or IGR, currently enrolled in a customized diet and/or training program, engagement in regular and vigorous physical activities and usage of medication affecting glucose balance (e.g., systemic corticosteroid medication).

Mean (±SD) subject age, weight, and BMI for the RT, NW, and C groups were as follows: 54 (±6.2), 56 (±5.6), and 54 (±6.9) years, 98 (±10.4), 94 (±9.8) and 90 (±11.5) kg, 31 (±3.1), 30 (±3.8) and 29 (±3.2) kg/m^2^, respectively. For detailed description of blood sampling, physical measurements and clinical analyses see Venojärvi et al., fitness and metabolic parameters are shown in Table 1 (Venojärvi et al. 2013b). In brief, blood samples were drawn after an overnight fast (12 h) from the brachial vein of each subject before and after 12 weeks. A 2‐h OGTT with 75‐g of glucose loading was also performed before and after the intervention period. Serum and glucose tubes were centrifuged at 2200***g*** for 10 min. Glycated hemoglobin (HbA_1c_) and glucose were analyzed using a Konelab 20i analyzer (Thermo Clinical Labsystems Oy, Konelab, Finland) and serum insulin was analyzed by chemiluminescence‐immunoassay. Homeostasis Model Assessment for insulin resistance (HOMA‐IR) was calculated as follows: HOMA‐IR = fasting serum insulin (*μ*U/mL) × fasting plasma glucose (mmol/L)/22.5 (Matthews et al. 1985).

**Table 1:** Fitness and metabolic parameters before and after 12 weeks of training

	Resistance			Nordic Walking			Control		
	Pre	Post		Pre	Post		Pre	Post	
VO_2_ peak (mL × kg^−1^ × min^−1^)	28.4 ± 1.1**#**	28.9 ± 1.1	*n* = 18, *n* = 17	29.2 ± 2.0**#**	31.0 ± 2.3*	*n* = 15, *n* = 14	34.5 ± 1.8	34.7 ± 2.1	*n* = 11, *n* = 11
UKK (index)	67.8 ± 5.1	78.3 ± 5.0*	*n* = 18, *n* = 18	76.3 ± 5.6	93.2 ± 5.7*	*n* = 17, *n* = 14	80.6 ± 4.2	91.7 ± 4.8*	*n* = 14, *n* = 14
Insulin (*μ*IU/mL)	12.7 ± 1.6**#**	12.4 ± 1.3	*n* = 20, *n* = 20	14.8 ± 2.1**#**	11.6 ± 1.4*	*n* = 18, *n* = 18	7.2 ± 1.1	8.6 ± 1.7	*n* = 16, *n* = 16
HOMA‐IR	3.5 ± 0.4**#**	3.3 ± 0.3	*n* = 20, *n* = 20	4.2 ± 0.6**#**	3.2 ± 0.4*	*n* = 18, *n* = 18	2.0 ± 0.3	2.3 ± 0.5	*n* = 16, *n* = 16
HbA _1c_ (%)	5.5 ± 0.1	5.5 ± 0.1	*n* = 19, *n* = 20	5.5 ± 0.1	5.5 ± 0.1	*n* = 17, *n* = 18	5.4 ± 0.1	5.7 ± 0.1*	*n* = 16, *n* = 16
2‐h glucose (mmol/L)	6.5 ± 0.4	6.0 ± 0.4	*n* = 18, *n* = 18	6.9 ± 0.6	6.3 ± 0.5	*n* = 14, *n* = 14	5.8 ± 0.4	5.6 ± 0.4	*n* = 16, *n* = 16

*P ≤* *0.05, compared to Pre [pairwise comparison (LSD) within groups]; ^#^ *P *≤* *0.05, compared to the C group [One‐way ANOVA (LSD) baseline values between groups]; ^§^ *P *≤* *0.05, compared to the RT group [One‐way ANOVA (LSD) baseline values between groups]; ^&ddagger;^ *P *≤* *0.05, compared to the NW group [One‐way ANOVA (LSD) baseline values between groups]. Values are presented as mean ± SEM.

#### Exercise intervention

Training in both intervention groups was performed three times (60 min/session) per week for 12 weeks. The exercise intensity and load were increased progressively after every 4 weeks of training.

The resistance training started with warm‐up exercises including cycling or rowing with an ergometer for 5 min and then stretching of the main muscle groups. In brief, exercise was performed using regular resistance equipment such as machines, dumbbells, and barbells. The program included leg press, bench press, leg extension, lateral pull‐down, leg flexion and shoulder flexion, explosive leg squats, squat jumps, standing calf jumps, or heel raises. Push‐ups, abdominal flexion, and back extension were performed without external weight. External loads started at 50% of exercise‐specific maximal strength (predetermined by 5RM test according to ([−4.18 × RM‐value of load] + 103) (McDonagh and Davies 1984) and reached 85% by week nine, which was sustained until the end of the 12th week (week [w] 1–2 at 50% 2 × 10 repetitions [reps], w 3–4 at 60% 3 × 5 reps, w 5–7 at 70% 3 × 5 reps, w 8–9 at 80% 3 × 5 reps, w 10–12 at 80–90% either 3 × 3, 1 × 3 reps or 3 × 5 reps depending on exercise). Training progression was controlled by 5RM strength measurements during the seventh training week. At the end of every session, subjects cooled down by low intensity cycling or rowing with the ergometer for 5 min and by stretching the main muscle groups. Before the NW intervention began, subjects were familiarized with using the poles in a safe and efficient way. All sessions started with a 5 min warm‐up exercises (400–500 m walking) and stretching of the main muscle groups. The aerobic exercise sessions were carried out at intensity levels increasing from 55 to 75% of heart rate reserve [w 1–4 at 55%, w 5–8 at 65%, and w 9–12 at 75%]. Individual target heart rates were calculated by using measured resting heart rate and the maximal heart rate estimated with the formula [210 – (0.65 age in years)]. Heart rate was monitored during training with Polar F4 (Polar Electro Oy, Kempele, Finland) heart rate monitors and the target heart rate range was progressively increased. To achieve the desired heart rate target, subjects either increased their walking speed or added uphill walking. After the session the main muscle groups were stretched during 5 min cool‐down.

All intervention programs were individually designed and supervised. Participants were advised not to change their habitual diet or lifestyle during the intervention.

### Biopsy protocol

Muscle biopsies from the vastus lateralis muscle were obtained at rest before the exercise intervention and 48–72 h after the last training session using the percutaneous needle biopsy technique (Bergström 1962). All biopsy samples were immediately frozen in liquid nitrogen and stored at −80°C until analysis.

### Protein extraction and ELISA

Muscle samples (approximately 20 mg) were homogenized in RIPA buffer (20 *μ*L RIPA/mg tissue) containing: 100 mmol/L NaCl; 50 mmol/L Tris Base; 5 mmol/L EDTA (pH 7.4); 0.5% Na deoxycholate; 0.1% SDS, 1% Triton‐X100; 1× complete protease inhibitor cocktail and 1× PhosStop (Roche Diagnostics, Mannheim, Germany), using a bead homogenizer, Retsch MM401. The homogenate was gently rotated at 4°C for 1 h, followed by centrifugation at 4°C for 10 min (15,000***g***). The protein content was determined using Bradford assay with BSA as standard. Samples were stored at −80°C until further use.

To measure protein levels of HN, skeletal muscle homogenates (100 *μ*g protein/well), and 100 *μ*L of 1:2 dilution of serum were loaded on a competitive ELISA (MyBioSourse #MBS744343, San Diego, CA) plate in duplicates and processed according to the manufacturer's instructions. Plates were scanned using a Microplate Photometer (Thermo Scintific Muliscan FC, Shanghi, China) with a 450 nm filter.

### RNA extraction

Total RNA from the skeletal muscle biopsies was prepared (from approximately 15 mg of tissue), using an acid phenol method (Chomczynski and Sacchi 1987) and quantified spectrophotometrically (NanoDrop^®^ 2000; Thermo Scientific, Gothenburg, Sweden). One microgram of total RNA was reverse transcribed according to manufacturer's instruction using the High Capacity Reverse Transcription Kit (Applied Biosystems, Foster City, CA) in a total volume of 20 *μ*L. Samples were stored at −80°C until further use.

### Real‐Time RT‐PCR

Real‐time RT‐PCR was used for mRNA quantification of mitochondrially encoded 16S rRNA (MT‐RNR2), encoding the HN peptide and the full length of ribosomal RNA 16s, forward primer: AATCACTTGTTCCTTAAATAGGGACC, reverse primer: GAACCCTCGTGGAGCCATT, and nuclear encoded humanin like ‐1 (MT‐RNR2L1), forward primer: CACTTGTTCCTTAAATAGGGACTTGTC, reverse primer: AGCTGAACCCTCGTGGAGC (Bodzioch et al. 2009). Glyceraldehyde‐3‐phosphate dehydrogenase (GAPDH) was used as an endogenous control, and proved stable across all time points. Primers were designed within an exon or to cover exon–exon boundaries to avoid amplification of nuclear DNA. All primers were synthesized by Eurofins Genomics, Ebersberg, Germany. The total reaction volume was 10 *μ*L, containing: 2 *μ*L cDNA sample; primer forward (final concentration 0.3 *μ*mol/L); primer reverse (final concentration 0.3 *μ*mol/L); and SYBR Green PCR Master Mix (Applied Biosystems). All quantification reactions were controlled with a melting curve and primer efficiency was tested using standard curves. For quantification of the mitochondrial encoded 16S rRNA (MT‐RNR2) (HS02596860) and mitochondrial encoded 12S rRNA (MT‐RNR1) (Hs02596859), the TaqMan Gene Expression Assays (Applied Biosystems) were used. The total reaction volume was 10 *μ*L, containing: 2 *μ*L cDNA sample; 5 *μ*L TaqMan Fast Universal PCR Master Mix (Applied Biosystems); and 0.5 *μ*L gene‐specific primers. GAPDH was used as an endogenous control (4352934E, Applied Biosystems), and it was stable across all time points. All reactions were performed in 384 well Hard‐Shell PCR plate (#HSP3951, Bio Rad, Hercules, CA), with sample duplicates, using the Bio Rad CFX384 Real Time System, C100 Touch Thermal Cycler. MTRNR2 mRNA was analyzed both with SYBR Green and TaqMan with equal results, TaqMan data are displayed in figure 2.

### Citrate synthase activity

The assay was performed according to the fluorometric principles of Lowry & Passonneau (1972) (Lin et al. 1988). In brief, a section of a biopsy was homogenized in 0.1 mol/L phosphate buffer (pH 7.7) with 0.5% BSA. For citrate synthase (CS), wet tissue lysates were added to a reagent solution (0.1 mol/L Tris‐HCl, 2.5 mmol/L EDTA, 0.5 mmol/L l‐malate, 512.5 nmol/L NAD^+^, 399 *μ*g MDH). 50 *μ*g acetyl‐CoA started the reaction and the velocity was registered with a fluorometer (reduction in NAD^+^ to NADH). A standard curve computed from known amounts of NADH was subsequently used to determine the CS activity. Correction for wet muscle weight was performed (Sahin et al. 2006; Vigelsø et al. 2014). Due to limited material, CS activity could only be analyzed in the RT group (*n *=* *14) and the C group (*n *=* *11).

### DNA extraction

DNA was isolated with a modification of the Gentra Puregene Tissue Kit (Qiagen, cat.# 69504, Germantown, MA). In brief, 5–10 mg skeletal muscle tissue was homogenized in lysis buffer (Puregene cat.# D‐5002) with proteinase K (Qiagen cat.# 19131), and incubated at 55°C for 1 h. Protein was precipitated followed by centrifugation 3 min at 14,000***g***. DNA was subsequently precipitated using ice‐cold isopropanol. DNA was washed in 70% EtOH and mixed with 50 μL of DNA hydration solution (Puregene cat.# D‐5004). After incubation at 65°C for 1 h, the concentration and quality of the DNA was analyzed using a Nanodrop^®^ 2000 spectro‐photometer (NanoDrop 2000; Thermo Scientific, Gothenburg, Sweden).

### Real‐Time RT PCR for mitochondrial DNA and nuclear DNA copy number

Total DNA was isolated as described above. Analysis were performed in 384 well Hard‐Shell PCR plate (#HSP3951, Bio Rad), with sample duplicates, using the Bio Rad CFX384 Real Time System, C100 Touch Thermal Cycler. DNA was diluted to a concentration of 10 ng/μL and for mtDNA analysis samples were diluted 1:10,000. The total reaction volume was 10 *μ*L, containing: 2 *μ*L DNA sample; primer forward (final concentration 0.3 *μ*mol/L); primer reverse (final concentration 0.3 *μ*mol/L); and SYBR Green PCR Master Mix (Applied Biosystems). Cycle parameters: one cycle of 95°C for 3 min, followed by 40 cycles at 95°C for 10 sec and at 60°C for 30 sec, followed by one cycle 95°C for 10 sec, 65°C for 5 sec, and lastly 95°C for 5 sec. The human nuclear DNA was analyzed by measuring the Myogenin promotor (forward primer: AGGTGCTGT CAGGAAGCAAGGA, reverse primer: TAGGGGGAGGAGGGAACAAGGA) and mitochondrial DNA was analyzed measuring mitochondrially encoded cytochrome c oxidase I gene (COX1, forward primer: CCCCTGCCATAACCCAATACCA, reverse primer: CCAGCAGCTAGGACTGGGAGAGA) (Rabøl et al. 2009). Mitochondrial DNA content per genome was then calculated as the ratio of the mtDNA and the genomic DNA for each sample.

### Statistics

Gene expression and total protein abundance in response to 12 weeks of training were analyzed using linear mixed models (LMM) with factors time (Pre and Post) × group (exercise and control). If interactions were significant, the analyses were followed by pairwise comparison within groups using Fisher's least significant difference (LSD) and confidence interval adjustment with Šidák correction. The expression of each target gene was determined using 2^ΔΔCT^, which provides the level of expression of the target gene relative to the level of expression of the endogenous control (GAPDH) in each sample. Changes between groups regarding fitness and metabolic parameters were analyses with one‐way ANOVA. Change in CS activity between RT and C group were tested with student's *t*‐test. Associations between fitness and metabolic variables were assessed by the Pearson's correlation test at baseline and compared to both HN baseline and ΔHN. All statistical analyses were performed using SPSS version 23 (IBM SPSS, Chicago, IL). Analysis for outliers, defined as observations <Q1‐(1.5 × IQR) or >Q3 + (1.5 × IQR) were done, no outliers needed to be excluded from the analysis. Data were normally distributed and results are presented as mean ± SEM.

## Results

### Humanin protein levels

**Figure 1:**
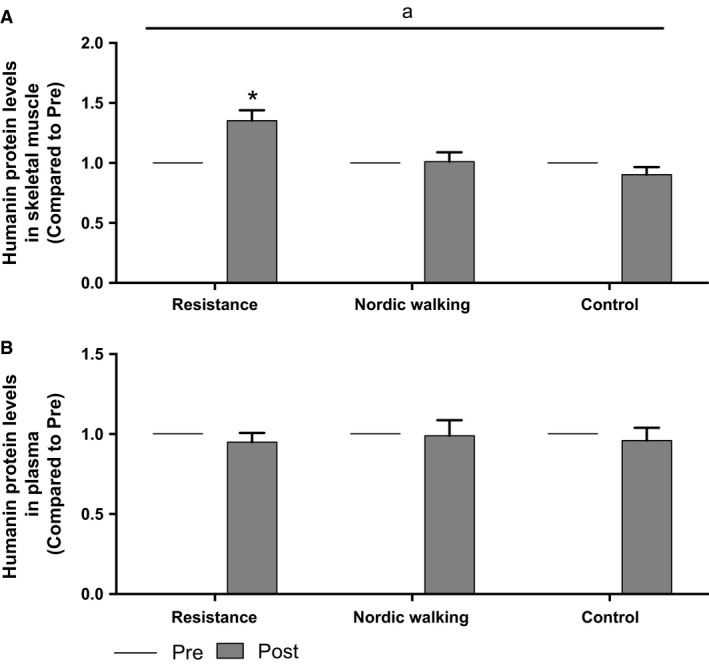
(A and B) Protein levels of Humanin in human skeletal muscle before and after 12 weeks of training (A), RT *n = *15, NW *n = *14, and C *n = *13. Protein levels of Humanin in human serum before and after 12 weeks of training (B), RT *n = *15, NW *n = *11, and C *n = *14. ^a^ *P* ≤ 0.05 with factors time × group (LMM). *P ≤* *0.05, compared to Pre [pairwise comparison (LSD) within groups]. Values are presented as mean ± SEM.

Skeletal muscle humanin (HN) protein levels increased significantly after 12 weeks of resistance training (1.35‐fold ± 0.085 (pre: 84.71 ± 4.9 pg/mL and post: 111.75 ± 6.85 pg/mL), *P* < 0.05, time × group interaction *P* = 0.022, Fig. 1A). No significant interactions over time were seen in the NW or the C groups (*P* > 0.05). Differences in HN baseline values were seen between the groups, with higher values in the NW and C group compared to the RT group (*P* < 0.05) and were accounted for as a covariate in the statistical analysis. Regression analysis of skeletal muscle revealed a weak but significant correlation (*R* = 0.311, *P* = 0.045) between HN at baseline and HOMA‐IR when grouping all subjects but not within groups. Humanin was also analyzed in serum but there was no significant change over time or between the groups (Fig. 1B). Regression analysis of the parameters age, VO_2_‐peak, UKK, Insulin and HbA_1c_ did not reveal any interactions or linear relationships in any of the three groups or when subjects were pooled, neither in skeletal muscle nor in serum. ΔHN serum levels in the RT group was significantly correlated with improvements in the 2 h glucose loading test (*R* = 0.597, *P* = 0.019). Blood metabolic markers and fitness parameters have been previously published for the larger randomized intervention study (*n* = 144) (Venojärvi et al. 2013a,b; Wasenius et al. 2014), however, this is the first investigation of skeletal muscle tissue from that intervention study.

### Humanin mRNA levels

**Figure 2:**
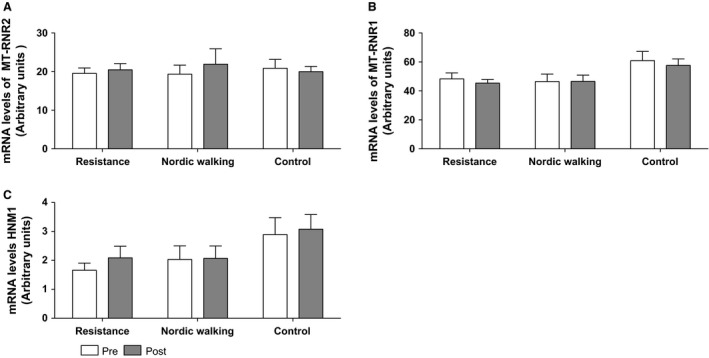
(A–C) mRNA levels of MT‐RNR2 (A), MT‐RNR1 (B), and HNM1 (C) in human skeletal muscle before and after 12 weeks of training. No significant change was detected. (A–C) RT *n = *17, NW *n = *12, and C *n = *16. Values are presented as mean ± SEM.

Twelve weeks of either RT or NW did not change the mRNA expression levels of the 16S gene (MT‐NRN2). There was no change over time or between the groups (NW, RT, or C, *P* > 0.05; Fig. 2A). There was no time x group interaction, neither in the intervention groups nor in the control group, for mRNA expression of MT‐NRN1, coding for the 12S gene, or of HNM1, the nuclear‐encoded humanin like‐ 1 gene (Fig. 2B and C).

### Mitochondrial markers

**Figure 3:**
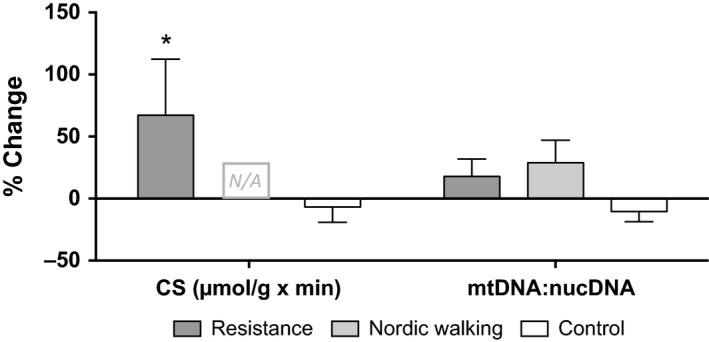
Left, CS enzyme activity as relative change (%) after 12 weeks’ intervention. RT *n *=* *14, NW = N/A, C *n = *11. Right, ratio between mtDNA and nuclear DNA displayed as relative change (%), RT *n = *15, NW *n = *15, and C *n = *15. Values are presented as mean ± SEM.

There was a significant increase in CS activity in the RT group (1.8‐fold ± 0.47 *P* < 0.05, time x group interaction *P* = 0.027). In the C group there was a decrease by 7%, however, not significant (Fig. 3). There was a nonsignificant increase by 18% in the ratio of mtDNA:nucDNA in the RT group (*P* = 0.37), a 29% increase in the NW (*P* = 0.27) group and a decrease of 10% in the control group (*P* = 0.09) see Figure 3.

## Discussion

In this study we show, for the first time, that a 12‐week resistance training intervention increases human skeletal muscle protein levels of humanin (HN) in a male population with impaired glucose regulation (IGR). It has previously been shown that resistance training interventions lasting 1–4 months lead to improved glucose metabolism and tolerance (Craig et al. 1989; Ishii et al. 1998; Cauza et al. 2005). The observed training‐induced increase in HN protein levels in skeletal muscle in this population is highly interesting since an HN analogue has previously been shown to increase glucose‐stimulated insulin secretion in both normal and diabetic mice (Kuliawat et al. 2013). Likewise, the improved glucose tolerance (Δ2 h) in the RT group correlated with changes in serum HN levels, indicating an endocrine rather than auto/paracrine effect of HN. This is in concordance with previous data from animals, showing that central administration of HN in rats results in elevated skeletal muscle glucose uptake and that IV injections of HN analogs lowers blood glucose in a rat model of diabetes (Muzumdar et al. 2009).

Taken together, previous findings and the present data strengthen the rationale to further study HN as a target in the prevention and treatment strategies for type 2 diabetes. Also, as implicated before in rodents, HN seems to have a role in the prevention of age‐related diseases such as type 2 diabetes and Alzheimer's disease (Muzumdar et al. 2009), and potentially also against cardiovascular diseases (Muzumdar et al. 2010; Zacharias et al. 2012; Zhang et al. 2012). However, any potential exercise‐mediated protective and risk reducing effects need to be further validated in future studies.

Resistance training, as well as endurance training, is known to activate AMPK (Dreyer et al. 2006; Lundberg et al. 2014). The initial discovery of HN led to the identification of another short peptide encoded within the mitochondrial 12S rRNA gene, the mitochondrial open reading frame of the 12S rRNA type‐c (MOTS‐c) (Fuku et al. 2015). Recently, MOTS‐c has been shown to be important for glucose regulation by activating AMPK and GLUT 4 in mouse skeletal muscle (Lee et al. 2015). Furthermore, MOTS‐c levels are reduced in mice suffering from obesity and insulin resistance (Lee et al. 2015). It may be speculated that MOTS‐c is involved in exercise adaptation (Lee et al. 2016) and the similarities between HN and MOTS‐c indicate that they might act in concert since the signaling pathways for HN are similar to those of MOTS‐c (Lee et al. 2013).

In this study, there was no significant increase in HN protein levels with endurance type Nordic walking training (NW). We speculate that the absence of any significant change in the NW group is due to the rather low exercise intensity conducted and not the exercise type per se. We also observed higher baseline values of HN in the NW group which could not be explained by differences in age, VO_2_‐peak, HOMA‐IR, Insulin levels, or HbA_1c_. It is possible that this might blunt a possible exercise‐induced increase in HN. We know that different types of exercise lead to different types of adaptations (Ingjer 1979; McDonagh and Davies 1984) and it has been debated whether concurrent endurance and strength exercises, through activation of partly different pathways, could interfere with each other and attenuate each specific training response (Baar 2006; Hawley 2009). However, resistance training has been shown to also activate molecular pathways usually coupled to endurance training (Sale et al. 1990; Wang et al. 2011) and a combination of resistance and endurance training has been shown to be more beneficial for the treatment of type 2 diabetes (Sigal et al. 2007).

Recently Voigt & Jelinek published a paper showing that patients with impaired fasting glucose (IFG) had decreased levels of humanin protein in plasma compared to a healthy control group (Voigt and Jelinek 2016). In our study, we did not compare our baseline values of the humanin peptide to healthy controls since we were only interested in a potential training response. Our data did not demonstrate any clear link between increased skeletal muscle HN protein and improved glucose metabolism in response to resistance training. On the contrary, only the NW group improved insulin sensitivity without any significant effects on HN protein levels. When data from the two intervention groups were pooled, the skeletal muscle HN protein level changes did not correlate to improvements in Hb1Ac, other metabolic markers or fitness parameters in our study. However, ΔHN levels correlated with improved glucose tolerance in the RT group in serum but not in skeletal muscle. Analysis of ΔHN levels in skeletal muscle and in serum did not correlate in any of the groups (data not shown). This inconsistency between tissues might be an effect of differences in protein turnover or indicate an endocrine regulation of HN since skeletal muscle is the main glucose‐handling tissue. It is also worth mentioning that both training response and protein levels in humans have a high individual variability. Nevertheless, the novel finding that resistance training increases the levels of HN in skeletal muscle in a population with IGR is highly interesting and may indicate that HN plays a role in exercise‐induced improvements of glucose regulation.

Unlike Voigt & Jelinek, we were unable to detect a correlation between HN levels and age. This could be explained by their pooling of data from the total cohort, including both healthy individuals and patients with IFG (Voigt and Jelinek 2016). In addition, our study only included males, whereas most subjects were females in the aforementioned study. Thus potential gender differences has to be taken into account (Lindholm et al. 2014). Furthermore, baseline BMI differences between these two study populations might play a role, with this study population having a higher BMI than the population by Voigt & Jelinek.

Increases in mitochondrial number and volume as well as biochemical changes within the mitochondria are well‐established responses to aerobic exercise training (Howald et al. 1985). Muscle mitochondrial density has been reported both in rodents and humans to increase between 50 and 100% after endurance training (Hood 2001). According to Larsen et al.*,* mtDNA is a modest biomarker of mitochondrial content, whereas CS activity displays a strong association (Larsen et al. 2012). Nevertheless, the mtDNA:nucDNA ratio as a marker for mitochondrial content has been evaluated and used previously (Sparks et al. 2005; Swerdlow et al. 2006; Guo et al. 2009; Rabøl et al. 2009; Tong et al. 2011). We report a significant increase in CS activity after 12 weeks of resistance training. Due to limited biopsy material availability in the NW group, we could only measure CS activity in three subjects and therefore measured the mtDNA:nucDNA ratio as an indication of oxidative adaptations in all groups. The observed trends toward higher mtDNa:nucDNA ratio in both intervention groups strengthens the suggested training effect and the CS enzyme activity seen.

The absence of any training‐induced changes in HN mRNA expression in this study might be due to the timing of the postbiopsy (48–72 h after the last training session). We might have missed mRNA expression changes that occur at time points closer to the previous exercise session (Perry et al. 2010; Gidlund et al. 2015). The posttraining biopsy time‐point was selected with the main purpose of studying long‐term training effects of the basal protein and mRNA concentrations. Since HN is encoded from a gene within a gene it is challenging to measure the mRNA coding for the HN peptide and not the 16S rRNA gene transcript. Therefore, we measured both the mitochondrially encoded genes MT‐RNR2 (containing the ORF coding for humanin) and MT‐RNR1 and the humanin‐encoded mitochondrial DNA‐like sequence (HNM1) in the nucleus (a NUMT) to assess whether they responded differently to training (Fig. 2) (Bensasson 2001; Mishmar et al. 2004; Richly and Leister 2004; Bodzioch et al. 2009). Although most NUMTs are considered pseudogenes, bioinformatics‐based evidence suggests that at least some of the nuclear MT‐RNR2‐like sequences might be functional genes (Bodzioch et al. 2009). Interestingly, these HN‐like genes are dispersed in multiple copies throughout the human genome, with HN‐like 1 (HNM1) being the one mostly expressed in skeletal muscle (Bodzioch et al. 2009). The function and regulation of these HN‐like genes have not been extensively studied. We did not detect any changes in HNM1 mRNA in the intervention groups over time. Even though not significant, there was a trend toward higher levels of HNM1 in the control group both at baseline and after the intervention period. It can be speculated that levels of HNM1 are elevated in more fit individuals since the control group differed at baseline compared to the intervention groups (VO_2_‐peak *P* < 0.05, weight *P* < 0.05, BMI *P* = 0.056 at baseline). However, the question whether HN is synthesized within the mitochondria or can be translated from the mitochondrial mRNA on cytoplasmic ribosomes remains to be answered. Future studies could benefit from measuring MT‐RNR2 after acute exercise to be able to separate elevated humanin expression from increased 16S rRNA gene expression (mitochondrial ribosome biogenesis) and mtDNA replication, and to focus on mechanistic studies of the HN ORF.

In conclusion, we show that skeletal muscle humanin (HN) protein levels increase with resistance training in the prediabetic state. Since HN is the first discovered mitochondrial‐derived peptide (MDP) that can act as a retrograde signaling molecule it is highly motivated to further investigate the role of this factor in training adaptations and in the prevention of type 2 diabetes through exercise.

## Conflict of Interest

None declared.
